# High Resolution Esophageal Manometry in Patients with Chagas Disease: A Cross-Sectional Evaluation

**DOI:** 10.1371/journal.pntd.0004416

**Published:** 2016-02-05

**Authors:** Adrián Sánchez-Montalvá, María Moris, Marianela Mego, Fernando Salvador, Anna Accarino, Kathleen Ramírez, Fernando Azpiroz, Antonio Ruiz-de-Leon, Israel Molina

**Affiliations:** 1 Infectious Diseases Department, Tropical Medicine Unit, PROSICS (International Health Program of the Catalan Health Institute), Vall d’Hebron University Hospital, Universitat Autònoma de Barcelona, Barcelona, Spain; 2 Digestive System Research Unit, Vall d'Hebron University Hospital, Centro de Investigación Biomédica en Red de Enfermedades Hepáticas y Digestivas (Ciberehd), Department of Medicine, Universitat Autònoma de Barcelona, Barcelona, Spain; 3 Department of Radiology, Vall d’Hebron University Hospital, Barcelona, Spain; 4 Department of Gastroenterology, University Hospital San Carlos, Universidad Complutense, Madrid, Spain; Institute of Tropical Medicine (NEKKEN), JAPAN

## Abstract

**Introduction:**

Gastrointestinal involvement affects 30–40% of the patients with chronic Chagas disease. Esophageal symptoms appear once the structural damage is established. Little is known about the usefulness of high resolution manometry to early identification of esophageal involvement.

**Method:**

We performed a cross-sectional study at the Vall d’Hebron University Hospital (Barcelona, Spain) between May 2011 and April 2012. Consecutive patients diagnosed with Chagas disease in the chronic phase were offered to participate. All patients underwent a structured questionnaire about digestive symptoms, a barium esophagogram (Rezende classification) and an esophageal high resolution manometry (HRM). A control group of patients with heartburn who underwent an esophageal HRM in our hospital was selected.

**Results:**

62 out of 73 patients that were included in the study fulfilled the study protocol. The median age of the Chagas disease group (CG) was 37 (IQR 32–45) years, and 42 (67.7%) patients were female. Twenty-seven (43.5%) patients had esophageal symptoms, heartburn being the most frequent. Esophagogram was abnormal in 5 (8.77%). The esophageal HRM in the CG showed a pathological motility pattern in 14 patients (22.6%). All of them had minor disorders of the peristalsis (13 with ineffective esophageal motility and 1 with fragmented peristalsis). Hypotonic lower esophageal sphincter was found more frequently in the CG than in the control group (21% vs 3.3%; p<0.01). Upper esophageal sphincter was hypertonic in 22 (35.5%) and hypotonic in 1 patient. When comparing specific manometric parameters or patterns in the CG according to the presence of symptoms or esophagogram no statistically significant association were seen, except for distal latency.

**Conclusion:**

The esophageal involvement measured by HRM in patients with chronic Chagas disease in our cohort is 22.6%. All the patients with esophageal alterations had minor disorders of the peristalsis. Symptoms and esophagogram results did not correlate with the HRM results.

## Author Summary

Chagas disease is a parasitic disease mainly transmitted to humans by blood-sucking insects. The disease was endemic in Latin America, but it is now a global disease due to migratory movements. The disease can affect the heart and the digestive system (mainly esophagus and colon). Classically, esophageal assessment in Chagas disease is performed by X-ray and self-reported symptoms. However, they lack accuracy and detect only advanced stage of the disease. Recently, new tools, such as esophageal high resolution manometry, provide more detailed information about the motility disorders of the esophagus. We assessed the esophageal involvement in patients with Chagas disease by means of high resolution manometry and compared the findings with the X-ray and self-reported symptoms. We found a low rate of mild severity motility disorders. We did not find an association between X-ray assessment and symptoms with the high resolution manometry findings. The assessment of esophageal involvement in patients with Chagas disease may benefit from early diagnosis by high resolution manometry, although more research is needed.

## Introduction

Chagas disease (CD) is caused by the hemoflagellate protozoan, *Trypanosoma cruzi*. CD is endemic in Latin America. However migratory flows have spread the disease all over the world; the non-endemic countries with more cases are The United States of America and Spain.[1] Its prevalence worldwide is around 10 million cases and 25 million people remain at risk.[2]

Patients with the chronic form of CD usually remain asymptomatic for years. Decades after the infection around 30–40% of the patients will develop visceral involvement caused by direct lesion, inflammation and fibrosis of the affected organs. The disease affects mainly the heart and the gastrointestinal tract, leading in some cases to dilated cardiomyopathy, megacolon and megaesophagus. [3]

Gastrointestinal involvement ranges between 5–35% of patients, showing lower frequencies in studies from non-endemic countries, despite being similar populations.[3,4] Dilatation of the digestive tract and motor disorders, such as achalasia, delayed gastric emptying and altered colonic transit, are found in affected patients.[5] The pathogenesis behind the digestive involvement lays on enteric nervous system injury.[6]

Dysphagia is usually the first symptom of esophageal involvement in CD, and achalasia and dilatation of the esophagus the main findings.[7] Contrary to what is seen in idiopathic achalasia, the pressure in the lower esophageal sphincter (LES) in patients with CD is diminished,[8] reflecting an impairment in both excitatory and inhibitory innervations. Unfortunately, the treatment for esophageal involvement in CD is addressed to symptoms relief.

Classically, esophageal assessment in CD is performed with barium swallow. Conventional manometry has also been used in research studies. Recently, new tools that provide a greater knowledge about the esophageal involvement such as high resolution manometry (HRM) are available; however data of their use in CD are still scant.

The objective of this study is to describe the findings of the esophageal HRM in patients with CD and to correlate esophageal symptoms with parameters and patterns of the esophageal HRM.

## Methods

### Study population

Consecutive CD patients attending the Tropical Medicine Unit of the Vall d’Hebron University Hospital (Barcelona, Spain) between May 2011 and April 2012 were invited to participate. Patients were referred to our units from blood donors centers, primary care centers, mother-to-child transmission prevention program and emergency service or hospitalization. Some patients came directly to other unit for a voluntary screening.

Inclusion criteria included a minimum age of 18 years old and confirmed diagnosis of CD in the chronic phase. Patients previously treated for Chagas disease, pregnant women and patients who declined to sign the informed consent were excluded. All subjects underwent ECG and chest X-ray. Treatment with benznidazole was offered to all patients after the esophageal assessment.

A control group was selected from patients with heartburn who underwent an esophageal HRM in our Hospital from April 2009 to April 2012. This control group was selected because heartburn was the most frequent symptom among the CD cohort. Patients in the control group were born in Spain (except for one patient that was from Equatorial Guinea) and had no risk factor for CD. All patients in the control group were surveyed using the same questionnaire as the CD group.

### Chagas disease diagnosis

Diagnosis of CD was based on two positive serological enzyme-linked immunosorbent assay (ELISA) tests, one with recombinant antigen (Bioelisa Chagas, Biokit, Lliçà d’Amunt, Spain) and the other with crude antigen (Ortho *T*. *cruzi* ELISA, Johnson & Johnson, High Wycombe, United Kingdom). Additionally, *T*. *cruzi* DNA was assessed in the peripheral blood by qualitative PCR before initiating treatment with benznidazole. PCR assay was performed according to Piron et al[9].

### Assessment of esophageal involvement

Rome III criteria were used to evaluate digestive symptoms through a personal interview. The interviewer had a structured questionnaire to guide the process.[10] The following items were included in the questionnaire: heartburn, dysphagia to liquids and solids, chest pain and regurgitation.

All patients underwent barium esophagogram and high resolution manometry. Results of the esophagograms were classified according to Rezende classification.[11]

### High resolution manometry

All the manometric studies were carried out with HRM, consisting in a solid-state catheter with 36 circumferential sensors, (each of them having 12 different entry ports), spaced by 1-cm distance along the intracorporal part of the catheter assembly (Sierra Scientific Instruments Inc., Los Angeles, CA, USA). A fasting of a minimum of 8 hours was required before the procedure. All the drugs that could interfere with the esophageal motility were discontinued. The catheter was introduced transnasally with the patient seated, until the most distally recording sensors were correctly placed in the stomach. Once positioned, the catheter was fixed in place by taping it to the nose. Subsequently, the patient adopted the supine position and the beginning of the protocol was postponed 1 minute approximately, to facilitate the relaxation and stabilization of the esophageal motility. The protocol started with the measurements of the basal sphincters’ pressure after a 30-second period. During this time, the patient was requested to breathe normally and not swallow. A minimum of 10 swallows of water of 5-ml each were then administered spaced by 30 seconds. As a supplemental test, a final multiple rapid swallowing was performed in every patient previously to the catheter removal. Manometric data were analyzed using dedicated software, (ManoView, Given Imaging, Yoqneam, Israel), following the indications reported by Pandolfino et al. [12] The esophagogastric junction (EGJ) was firstly characterized. The proximal and distal limits were identified as abrupt increased changes in the pressure relative to both the intraesophageal and the intragastric pressures. Once identified, the integrated relaxation pressure (IRP) was recorded. An IRP equal or lower than 15 mmHg was considered normal. The distal esophageal body contractions were analyzed by the generation of isobaric contour plots at 30 mmHg. The mean values of the variables: Basal upper esophageal sphincter (UES) pressure (normal value 34–104 mmHg), relaxing UES pressure (normal value <12 mmHg), contractile front velocity (CFV) (normal value <9.0 cm/s), distal contractile integrated (DCI) (normal range 450–8000 mmHg s cm) and distal latency (DL) (normal value >4.5 seconds) were recorded in each study. [13]

The overall motility pattern was firstly classified following the 2012 Chicago classification criteria and reviewed after the recent publication of the new guidelines. [14,15] Thereby, the main categories considered were disorders with EGJ outflow obstruction (achalasia types I, II and III and EGJ outflow obstruction), major disorders of peristalsis (distal esophageal spasm, jackhammer esophagus, absent contractility), minor disorders of peristalsis (ineffective motility and fragmented peristalsis) and normal esophageal motility.

### Interobserver agreement

To avoid observer-dependent bias, all the HRM reports were analyzed by two experimented gastroenterologists who were unaware of the symptoms of the patient. If any disagreement was encountered, it was brought to discussion and the final decision was decided in consensus.

### Statistical analysis

Data were analyzed with IBM SPSS Statistics software (v.21.0.0.0; IBM SPSS, Armonk, NY). The median and interquartile range (IQR) were calculated for quantitative variables. Frequencies and percentages were calculated for qualitative variables. Analysis was performed using Student’s t-test or Mann–Whitney’s U test for quantitative variables and Chi-square test or Fisher’s test for qualitative variables when appropriate. Tests were considered significant when the two-tailed p-value was <0.05.

### Ethical considerations

The study protocol was approved by the Ethical Review Board of Vall d’Hebron Hospital (Barcelona, Spain) and procedures were carried out in accordance with the ethical standards laid down in the Helsinki Declaration as revised in 2000. All patients signed the informed consent after a careful explanation of the study.

## Results

Initially, 73 patients with a confirmed diagnosis of CD were included in the study. Sixty two patients fulfilled the study protocol and were analyzed. The remaining 11 patients did not come to perform the HRM and declined to reschedule the test. Thirty patients were included in the control group.

In the CD group, the median age was 37 (IQR 32–45) years. Forty two (67.7%) patients were female. All the patients in the CD group were migrants from Latin American countries (60 (96.8%) patients were from Bolivia, one from Paraguay and one from Equador). When comparing baseline characteristic between the two groups, the control group was older (37 vs 51 years; p<0.001) and had more esophageal symptomatology (43.5% vs 93.3%; p<0.001) than the CD group. More information is shown in Table 1.

**Table 1:** Epidemiological and clinical data.

	Chagas group (n = 62)	Heartburn control group (n = 30)	P value
Sex, female	42 (67.7%)	23 (76.7%)	0.468
Age, years	37 (32–46)	51 (37–66)	<0.001
Years living in host country	6 (5–8)	NA	
Bolivian origin	60 (96.8%)	NA	
Positive *T cruzi* PCR n = 58	28 (45.2%)	NA	
Normal esophagogram n = 57	52 (83.9%)	NA	
**Symptoms**			
*Heartburn*	19 (30.6%)	28 (93.3%)	<0.01
*Dysphagia to liquids*	4 (6.5%)	10 (33.3%)	0.02
*Dysphagia to solids*	9 (14.5%)	12 (40%)	0.009
*Chest pain*	3 (4.8%)	14 (46.7%)	<0.001
*Regurgitation*	9 (14.5%)	17 (56.7%)	<0.001
*Any esophageal symptoms*	27 (43.5%)	28 (93.3%)	<0.001

Note: Data are reported as number (percentage) of patients and mean values (interquartile range).

The clinical questionnaire was carried out in all included patients. Almost half of the CD group (27 patients (43.5%)) was symptomatic. From the symptomatic patients with CD, none of them referred a disabling intensity in any of the symptoms. Overall, the most frequent symptom was heartburn, which was referred by 19 (30.6%) patients. This was largely followed by regurgitation and dysphagia to solids. Among patients in the control group, 28 (93.3%) patients had at least one symptom and 6 (20%) patients referred disabling symptoms. Clinical data are summarized in Table 1.

**Fig 1:**
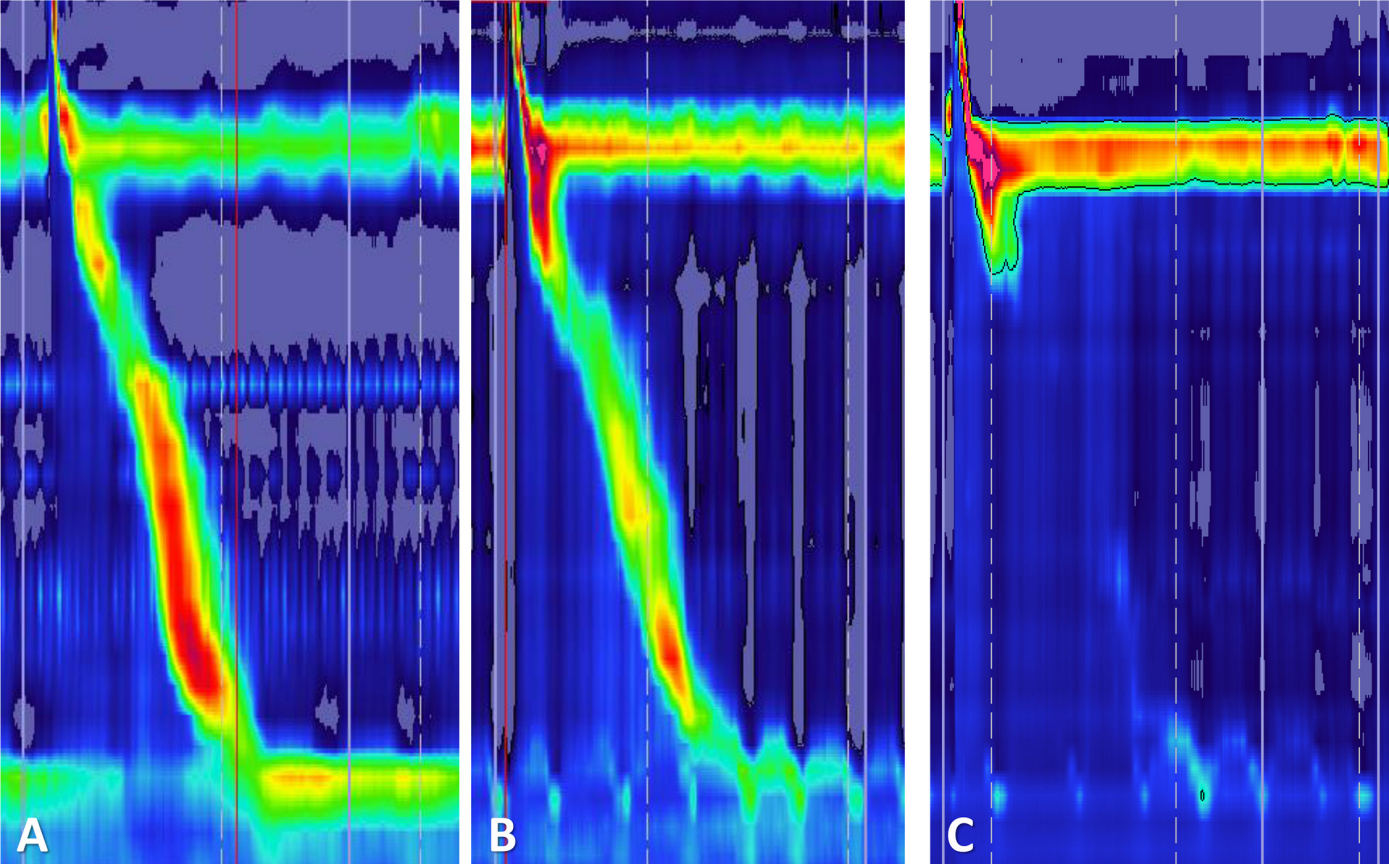
Examples of the high-resolution manometric studies in patients with Chagas disease: (A) Normal lower esophageal sphincter pressure and esophageal peristalsis (B) Hypotonic LES and normal peristalsis and (C) Ineffective esophageal motility and hypotonic LES. LES: lower esophageal sphincter.

The esophagograms were performed in 57 patients (92% of the CD group) being normal in the vast majority of the cases (52 patients (91.23%)). Only 5 (8.77%) patients had hiatus hernia. The esophagogram assessment was not performed in the control group. Fig 1 shows examples of HRM tracings.

A pathological motility pattern was seen in 14 (22.6%) patients in CD group. The HRM pattern more frequently found was minor disorders of the peristalsis (13 with ineffective esophageal motility and 1 with fragmented peristalsis). Hypotonic LES was present in 13 patients in the CD group, while in the control group 1 patient had hypotonic LES and 3 patients had hypertonic LES (p = 0.005). UES was normal in 39 (62.9%) patients, hypertonic in 22 (35.5%) and hypotonic in 1 patient in the CD group. In the control group 18 (60%) patients had a normal UES, 9 (30%) a hypertonic UES and 3 a hypotnic UES. More data can be found in Table 2 (HRM technical parameters) and in Table 3 (manometric patterns according to Chicago classification).

**Table 2:** Technical manometric parameters according to study group.

	Chagas group (n = 62)	Heartburn control group (n = 30)	P value
Basal LES pressure (mmHg)	7.2 (5–11.93)	14.65 (10.2–20.9)	**<0.001**
**Status Basal LES**			**0.005**
*Normal Basal LES pressure*	49 (79%)	26 (86.7%)	
*Hypotonic Basal LES pressure*	13 (21%)	1 (3.3%)	
*Hypertonic Basal LES pressure*	0	3 (10%)	
IRP (mmHg)	5.5 (51.75–77.75)	8.65 (5.03–10)	0.115
Normal IRP N: (<15mmHg)	62 (100%)	29 (96.7%)	0.326
CFV (cm/s)	3.1 (2.58–3.53)	3.6 (2.7–4.55)	0.071
Normal CFV (N: <9cm/S)	62 (100%)	28 (96.7%)	0.326
DCI (mmHg s cm)	978.25 (741.03–1657.58)	1279 (835.4–2199.35)	**0.014**
Normal DCI (N: 450–8000 mmHg s cm)	58 (93.5%)	28 (96.6%)	1
DL (seconds)	7 (6.28–7.63)	6.6 (5.6–7.2)	0.15
Normal DL (N: >4.5s)	62 (100%)	29 (100%)	NA
IBP (mmHg)	0.9 (-0.53–3.03)	21.1 (16.9–25.2)	**<0.001**
Basal UES pressure (mmHg)	86.75 (64.65-135-23)	74 (51.1–115.43)	0.078
Normal basal pressure UES (N: 34–104mmHg)	39 (62.9%)	18 (60%)	0.788
Relaxing UES pressure (mmHg)	3.75 (1.08–8.05)	6.15 (3.18–9.83)	**0.011**
Normal Relaxing pressure UES (N: <12mmHg)	58 (93.5%)	24 (80%)	0.073

Note: LES, lower esophageal sphincter; IRP, Integrated relaxation pressure; CFV, contractile front velocity; DCI, distal contractile integral; DL, distal latency; IBP, Intrabolus pressure; UES, upper esophageal sphincter. Data are reported as number (percentages) of patients and mean values (interquartile range).

**Table 3:** High resolution manometries results according to the Chicago classification in Chagas cohort and control group.

Classification	Chagas group (n = 62)	Control group (n = 30)	P value
**Normal**	48 (77.4%)	24 (80%)	0.776
**Achalasia or EGJ outflow obstruction**	0	0	NA
**Major disorders of peristalsis**	0	2 (6.7%)	.104
*Absent contractility*	0	0	
*Distal esophageal spasm*	0	1 (3.35%)	
*Hypercontractile esophagus*	0	1 (3.35%)	
**Minor disorders of peristalsis**	14 (22.6%)	4 (13.3%)	0.295
*Ineffective esophageal motility*	13 (21%)	4 (13.3%)	
*Fragmented peristalsis*	1 (1.6%)	0	

Note: Data are reported as number (percentages) of patients.

When comparing manometric parameters or patterns in the CD group according to any esophageal symptom no statistically significant association was seen, except for the median values in the DL parameter (7.2 symptomatic patients vs 6.5 non-symptomatic patients; p = 0.049), however all the patients were considered to have normal values. Data are depicted in Table 4.

**Table 4:** Relationship between the manometric findings and the symptoms in patients with Chagas disease.

	Absence of symptoms (n = 31)	Presence of the symptoms (N = 27)	P value
Normal HRM	23 (74.2%)	23 (85.2%)	0.303
Basal LES pressure (mmHg)	6.4 (4.2–11.5)	8 (5.2–13.2)	0.627
Normal Basal LES pressure	24 (77.4%)	22 (81.5%)	0.703
IRP (mmHg)	5.4 (3.4–9.5)	5.4 (4.2–7.7)	0.899
Normal IRP N: (<15mmHg)	31 (100%)	27 (100%)	NA
CFV (cm/s)	3.2 (2.4–3.6)	3.2 (2.8–3.7)	0.973
Normal CFV (N: <9cm/S)	31 (100%)	27 (100%)	NA
DCI (mmHg s cm)	1107.4 (769.2–1817.9)	899.4 (542.3–1557.7)	0.264
Normal DCI (N: 450–8000 mmHg s cm)	28 (90.3%)	26 (96.3%)	0.615
DL (seconds)	7.2 (6.4–7.7)	6.5 (5.9–7.3)	**0.049**
Normal DL (N: >4.5s)	31 (100%)	27 (100%)	NA
Basal UES pressure (mmHg)	98.5 (70–140)	83.1 (61.7–134.3)	0.271
Normal basal pressure UES (N: 34–104mmHg)	17 (54.8%)	19 (70.4)	0.224
Relaxing UES pressure (mmHg)	4 (1–8.9)	3 (1.6–7)	0.697
Normal Relaxing pressure UES (N: <12mmHg)	30 (96.8%)	25 (92.6%)	0.593

Note: HRM, high resolution manometry; LES, lower esophageal sphincter; IRP, Integrated relaxation pressure; CFV, contractile front velocity; DCI, distal contractile integral; DL, distal latency; IBP, Intrabolus pressure; UES, upper esophageal sphincter. Data are reported as number (percentages) of patients and mean values (interquartile range).

## Discussion

Our study describes the main esophageal HRM findings in a cohort of consecutive patients with chronic CD. It reports the presence of low-intensity esophageal symptoms in almost half of the patients in the CD group, but there was no association between esophageal symptoms and the findings in the esophageal HRM.

In CD the esophageal involvement is mainly characterized by the loss of the myenteric plexus. This translates in a motility pattern similar to that described in idiopathic achalasia, consisting in simultaneous contractions of low amplitude in the esophageal body, however LES in CD patients with esophageal involvement tends to be normal or hypotonic.[16,17] These alterations have been thoroughly studied with conventional esophageal manometry, however, few studies to date have reported the main pathological findings using HRM.

Herein, no specific inclusion criteria regarding the symptoms were applied to avoid selection bias, and therefore, obtain a representative sample of the chronic CD population. Thus, 56.5% of the cohort was completely asymptomatic. This percentage is consequent with current literature findings, reporting values that range between 33–52%.[18,19] These percentages cannot be interpreted as a lack of esophageal involvement due to CD as it has been described that symptoms do not arise until the neuronal damage is extensive, [20] and as our study shows the vast majority of the patients had a normal esophageal HRM despite low esophageal symptoms.

The first symptom usually developed in the course of esophageal involvement in patients with CD is dysphagia, however depending on the study the results may vary. [7] In our study, the most frequent symptom was heartburn, while dysphagia was present only in few patients. Heartburn in patients with CD is frequently accompanied by normal esophageal tests, while dysphagia goes normally with altered esophageal tests. Heartburn is a very common symptom in the community and its positive predictive value for esophageal involvement in patients with CD is low. This observation may be explained by the low esophageal involvement in our cohort and goes accordingly with previous studies. [18,21] When great damage is already established in the esophagus, heartburn gives way to dysphagia as the main symptom.

On the other hand, it should be mentioned that 10 patients of our study referred dysphagia (3 of them with dysphagia to both solid and liquids). In 9 of these cases, the barium esophagogram was strictly normal, (one patient did not have this test performed), and in 8 patients the HRM was informed as completely normal. The remaining 2 had minor disorders of the peristalsis which may be considered an early manifestation of CD esophageal involvement. Hence, it seems possible that dysphagia in these patients was not a consequence of CD as this symptom appears once there is a large damage of the esophagus.

The results of the diagnostic tests were also remarkable for the absence of significant pathological findings. None of the barium esophagograms showed any grade of dilatation, with the only abnormality being a hiatus hernia. Based on our findings, we suggest that barium esophagograms should not be performed as part of the initial evaluation of every patient with chronic CD, due to its lack of discrimination of early esophageal alterations.

HRM provides clear advantages with respect of conventional manometry in the characterization of the esophageal contractions and, more importantly, in the definition and features of the LES. [22] As a result, it has been postulated that the application of this technique in the evaluation of chronic CD would lead into the description of motility patterns that may have been unnoticed with conventional manometry. Surprisingly, in our cohort the motility alterations were remarkably mild. For instance, 77.4% of the sample had a complete normal HRM report. In the remaining patients, 14 patients had minor disorders of the peristalsis. When comparing with the heartburn control group, the basal pressure of the LES was lower in the CD group and the rate of hypotonic LES was also statistically lower. These findings are in concordance with current knowledge that states that the involvement of the esophagus of CD is due to destruction of the enteric nervous system, affecting both inhibitory and excitatory nerves [6].

There are four studies that assessed esophageal HRM in patients with chronic CD. All of them were performed in endemic countries. Two studies performed by Vicentine et al [23,24] in Brazil included patients with CD and dilatation of the esophagus and one study by Silva et al [25] also performed in Brazil selected patients with symptoms. These three studies cannot be compared with our study because their cohorts were selected among symptomatic patients or patients with achalasia-like esophagus. The remaining study by Remes-Troche et al [18] included 42 consecutive patients irrespective of their clinical symptoms. They found symptoms in 33% of the patients. The HRM assessment surprisingly showed that 28 (66%) of the patients had esophageal motility disorders according to the former Chicago classification. However, with the new Chicago classification it is probable that less esophageal abnormality would have been described. Our study also found that the most frequent abnormality is minor disorders of the peristalsis, however we did not find any patients with major disorders, EGJ outflow obstruction or achalasia. On the other hand, no statistically significant relationship was seen between the presence or absence of symptoms and the esophageal HRM findings in our cohort, similarly to previous studies. [21]

We acknowledge some limitations in our study. The limited sample size may have prevented us to find some significant associations between the symptoms and the manometric findings due to a possible lack of power. Moreover, a specific study with upper endoscopy or pH-metry was not performed as part of the study protocol to further characterize the heartburn referred by some of the patients. Additionally, the control group was not matched to the cohort with respect to origin and age, so some findings may be attributed to these factors.

To the best of our knowledge, this is one of the few studies to date using esophageal HRM in chronic CD and the first reporting this technique in a non-endemic country. We reported esophageal motility disorder in 22.6% of a cohort of consecutive CD patients. Symptoms and esophagogram results did not matched with the HRM results. We believe that further prospective studies are needed, especially with HRM, to elucidate the evolution of early abnormal motility patterns in chronic esophageal CD.

## Supporting Information

S1 Dataset Study data base. (SAV)

S1 Checklist STROBE checklist. (DOCX)
